# Identification of aspects of functioning, disability and health relevant to patients experiencing vertigo: a qualitative study using the international classification of functioning, disability and health

**DOI:** 10.1186/1477-7525-10-75

**Published:** 2012-06-27

**Authors:** Martin Mueller, Edith Schuster, Ralf Strobl, Eva Grill

**Affiliations:** 1 Institute for Medical Informatics, Biometry and Epidemiology, Ludwig-Maximilians-Universität München, Marchioninistr 17, 81377, Munich, Germany; 2 Integrated Center for Research and Treatment of Vertigo, Balance and Ocular Motor Disorders (IFBLMU), Ludwig-Maximilians-Universität München, University Hospital Munich, Marchioninistr. 15, 81377, Munich, Germany

**Keywords:** Vertigo (MeSH), Outcome assessment (Health Care) (MeSH), Qualitative research (MeSH), Classification (MeSH)

## Abstract

**Purpose:**

Aims of this study were to identify aspects of functioning and health relevant to patients with vertigo expressed by ICF categories and to explore the potential of the ICF to describe the patient perspective in vertigo.

**Methods:**

We conducted a series of qualitative semi-structured face-to-face interviews using a descriptive approach. Data was analyzed using the meaning condensation procedure and then linked to categories of the International Classification of Functioning, Disability and Health (ICF).

**Results:**

From May to July 2010 12 interviews were carried out until saturation was reached. Four hundred and seventy-one single concepts were extracted which were linked to 142 different ICF categories. 40 of those belonged to the component body functions, 62 to the component activity and participation, and 40 to the component environmental factors. Besides the most prominent aspect “dizziness” most participants reported problems within “Emotional functions (b152), problems related to mobility and carrying out the daily routine. Almost all participants reported “Immediate family (e310)” as a relevant modifying environmental factor.

**Conclusions:**

From the patients’ perspective, vertigo has impact on multifaceted aspects of functioning and disability, mainly body functions and activities and participation. Modifying contextual factors have to be taken into account to cover the complex interaction between the health condition of vertigo on the individuals’ daily life. The results of this study will contribute to developing standards for the measurement of functioning, disability and health relevant for patients suffering from vertigo.

## Background

Vertigo and dizziness are among the most common health problems in medical practice [1-5]. Vertigo and dizziness include both consequences of disease as well as definable disease entities such as benign paroxysmal positional vertigo, Meniere’s disease, or vestibular migraine. Irrespective of the various causes and underlying health conditions, vertigo and dizziness have significant impact on functioning and overall quality of life of the affected individuals To give some examples, the most common peripheral-vestibular disorder benign paroxysmal positioning vertigo – which is also the most frequent type of vestibular disorder – causes brief rotatory vertigo attacks, mainly triggered by rapid head movements, e.g. when turning around in the bed or lacing shoes. Central-peripheral disorders, which may be caused by brain ischemia, multiple sclerosis, or other permanent or transient brain lesions, can make vertigo attacks lasting from minutes to even weeks [6]. In addition, vertigo and dizziness are a considerable burden to economy and health care [4,7,8].

Precondition of effective management and treatment of potentially disabling conditions like vertigo is – besides careful diagnosis of the underlying condition –the assessment of outcomes which are relevant to the patient. This is not only important to monitor treatment effects but also to set goals and to plan therapy [6].

As summarized by Morris et al. [9] outcome measures in vertigo vary by the entities they are addressing,, such as subjective experience of disability or signs and symptoms. Most of them reflect either the patients’ or health professionals’ experience. As an example, the frequently used Dizziness Handicap Inventory [10] addresses the patients’ experience of the consequences of vertigo on daily living.

Most outcome measures are developed and validated on the basis of empirical findings or professional experience [11]. However, none of them refer to a common theoretical framework. A theoretical framework is fundamental for defining an outcome measurement and helps to ensure that the whole potential spectrum of issues is reflected [12].

In addition to the lack of theoretical foundation there is still no agreement on standards used to measure outcomes in patients with vertigo. This is illustrated by a recently published Cochrane Review in the field of vestibular rehabilitation which noted 15 different outcome measures of patients’ complaints in 21 studies [13].

**Figure 1:**
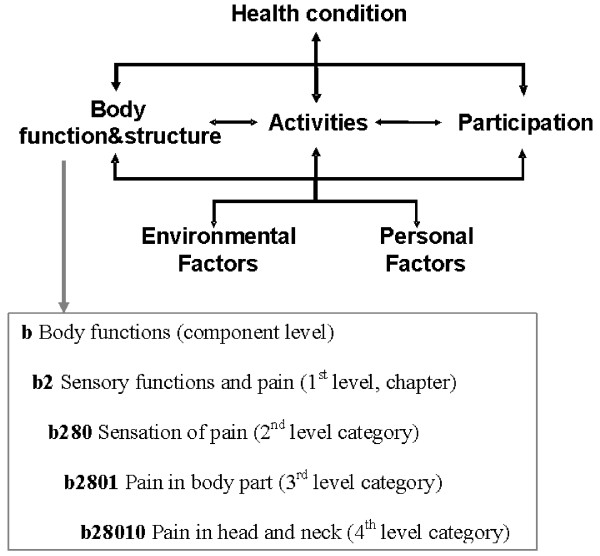
The ICF model of functioning, disability and health and an example of the hierarchical structure of the ICF.

To address both these issues, the Integrated Center for Research and Treatment of Vertigo, Balance and Ocular Motor Disorders (*IFB*^LMU^) at the Ludwig-Maximilians-Universität in Munich set up a project that aims to develop an international standard for the description of functioning and disability in patients with vertigo and dizziness based on the International Classification of Functioning, Disability and Health (ICF). With the ICF, which is part of the family of international classifications of the World Health Organisation (WHO), there is a common theoretical framework for describing and measuring health and disability. The ICF models the individuals' functioning and health as a complex interaction between a health condition and contextual factors. Additionally, the ICF classifies domains of functioning which are encountered in human life: Body structures and functions, activities and participation, along with their contextual Personal and Environmental Factors. The ICF classification contains more than 1400 hierarchically organized categories which describe the components of the ICF model in detail up to four levels (see Figure 1). One major goal of the ICF is to record and organize a wide range of information about health and health related states for individuals and populations. For the purpose of defining the contents of a comprehensive assessment, the ICF provides a universal language intended to be equally used and understood by health professionals and patients. Thus, it can be used to organize and standardize issues most relevant for patients with vertigo or dizziness while respecting patients' perspective and experiences. The ICF therefore has the potential to serve as a useful comprehensive basis to standardize health information on the individual and the population level in patient care and research [14,15].

The development of this international standard is based on the protocol to develop ICF Core Sets [16-18] and will therefore incorporate the perspectives of affected individuals, of health professionals, of current research as well as the epidemiological perspective.

The objective of this paper is to investigate the perspectives of patients with vertigo and dizziness on their experience of functioning and health using the ICF. Specific aims were

(1) to identify aspects of functioning and health relevant to patients with vertigo expressed by ICF categories and

(2) to explore the potential of the ICF to describe the patient perspective in vertigo.

## Methods

### Study design

We conducted a series of qualitative semi-structured face-to-face interviews using a descriptive approach [19]. The interviews were audio recorded and transcribed verbatim. The interviews were initiated as narrative interview. In the background, the interviewer had an interview guide that was designed to address the components of the International Classification of Functioning, Disability and Health (ICF): Body Functions, Body Structures, Activities and Participation, and the contextual factors Environmental and Personal Factors. The interview guide was developed using information of earlier focus groups and individual interview studies with the focus to explore relevant aspects of functioning and health in different populations [20,21]. The initial question and the optional questions on ICF components are presented in Table 1.

**Table 1:** Interview scheme

*Initial question*	When did vertigo/dizziness occur the first time? How many years ago?
*Body Functions*	If you think about the functions of your body, your mind and your soul, what does not work the way it is supposed to?
*Body Structures*	If you think about your body, in which parts are your problems?
*Activities and Participation*	If you think about your daily life, what are your problems? If you compare your life before the occurrence of vertigo/dizziness with your life now, what has changed?
*Environmental Factors*	If you think about your environment and your living conditions, what do you find helpful or supportive? If you think about your environment and your living conditions, what barriers do you experience?”
*Personal Factors*	If you think about yourself, what is crucial when handling your current situation?

### Additionally collected data

To describe the study sample, sociodemographic and disease specific data (age, sex, living situation, medical diagnosis) was collected. Vertigo was categorized as either central-vestibular vertigo, peripheral-vestibular vertigo, somatoform vertigo or combination of types. To describe an overall impression on participants’ health, they were asked to appraise their health on a 5-point-Likert scale ranging from one to five were one indicates best health. In addition, the impact of vertigo on health was appraised by the Dizziness Handicap Inventory (DHI) [10] and the Vertigo Symptoms Scale (VSS)[11]. The DHI addresses self-perceived handicap due to vestibular disorders. The score ranges from 0 to 100, with a higher score indicating greater handicap. The VSS addresses frequency and severity of dizziness symptoms within the last 12 months. The score ranges from 0 to 4, with a higher score indicating more frequent and more severe symptoms.

### Participants

Patients were recruited at the outpatient dizziness clinic at the Integrated Center for Research and Treatment of Vertigo, Balance and Ocular Motor Disorders (*IFB*^LMU^) at the Ludwig-Maximilians-Universität in Munich. Potential participants were contacted and asked for their willingness to contribute to a study by their physician in charge. In case of preliminary consent, the patients were provided with detailed information about the study. Informed written consent had to be signed prior to the beginning of the interview.

Inclusion criteria were over 18 years of age and adequate command of the German language. Positive vote of the ethics committee of the Medical Faculty of Ludwig-Maximilians-Universität in Munich was obtained prior to start.

The interviews took place subsequent to the consultation in the outpatient clinic in rooms of the hospitals. The interview was taken by an experienced registered nurse and bachelor student (EB) who was especially trained and ongoing supervised by the senior researchers (EG & MM). The interviewer was not part of the health care team.

### Sample size

The sample size was determined by saturation. Saturation refers to the point at which an investigator has obtained sufficient information from the field [22]. In this study we defined saturation as the point during data collection and analysis when an interview revealed less than 5% additional ICF categories. The sampling strategy adopted the idea of theoretical sampling from the grounded theory methodology [23]. In the selection process the researchers tried to balance relevant characteristics of the patients, such as gender, age and disease, to assure maximum sensitivity and to ensure a maximum of variety of experiences from the participants with different types of vestibular disorders to address those comprehensively.

### Data analysis

#### Qualitative data analysis

The meaning condensation procedure [24] was used for the analysis of data content. In the first step, the interview transcripts were read through to get an overview of incorporated meaningful concepts. In the second step, the text was divided into meaning units, and the dominating theme for this unit was determined. A meaning unit was defined as a specific unit of text either a few words or a few sentences with a common theme. In the third step, the specific concepts contained in the meaning units were identified. For quality assurance reasons, the qualitative data analysis was conducted independently by two researchers trained in the methodology (MM, ES). The results were compared and discussed prior to further analysis.

#### Linking to the ICF

The identified concepts were linked to the categories of the ICF based on established rules by two health professionals with expert knowledge of the ICF (MM, ES) [25]. According to linking rules, health professionals trained in the ICF are advised to attribute each concept to the ICF category representing this concept most precisely. One concept can be linked to one or more ICF categories, depending on the number of themes contained in the concept. Consensus between the two health professionals was required to decide which ICF category should be linked to each identified concept. In case of a disagreement, a third person trained in the linking rules was consulted. In a discussion led by the third person, the two health professionals that linked the concepts stated their pros and cons for the linking of the concept under question to a specific ICF category. Based on these statements, the third person made an informed decision. See Table 2 for a scheme of qualitative data analysis and linking.

**Table 2:** Scheme of qualitative data analysis and linking

**Interview text**	**Meaning unit**	**ICF category**
,,I had to quit working in (…).“	Quitting gainful employment	d850 Remunerative employment
„I can’t drive my car anymore, even to the next town.”	Unable to drive cars	d4751 Driving motorized vehicles

## Results

After 12 individual interviews from May until July 2010 saturation was reached. Characteristics of the participants are displayed in Table 3. Four hundred and seventy-one single concepts were extracted from the interviews. Those identified concepts were linked to 142 different ICF-categories describing relevant aspects of functioning in our patients. 40 of those ICF categories belonged to the component body functions, 62 to the component activity and participation, and 40 to the component environmental factors (see Table 4, 5 and 6). None of the concepts could be linked to categories out of the component body structures. Four percent of all extracted concepts could not be linked to specific ICF categories. Most of them were related to ‘personal factors’, specifically personal strategies of coping.

**Table 3:** Characteristics of the participants

	**age in yrs**	**gender**	**living situation**^**1**^	**Type of vertigo**^**2**^	**time with vertigo (in yrs.)**	**general health**^**3**^	**Vertigo Symptom Scale**^**4**^	**Dizziness Handicap Inventory**^**5**^
1	45	female	family	central	0.5	3	2.38	82.00
2	75	male	family	peripheral	20	4	1.00	18.00
3	69	male	family	peripheral	8	3	0.76	26.00
4	63	female	family	peripheral	2	3	-	22.00
5	67	male	family	peripheral	3	3	1.85	33.00
6	67	male	alone	combination	2	3	0.56	-
7	69	male	family	combination	0.5	4	1.18	32.00
8	75	female	family	peripheral	8	3	0.26	6.00
9	43	male	alone	somatoform	0.5	4	0.94	23.00
10	42	male	family	peripheral	0.5	4	-	-
11	29	female	family	central	1	4	-	-
12	53	female	family	combination	20	3	2.12	16.00

^1^alone = living alone, family = living in a household together with family or partner.

^2^central = central-vestibular vertigo, peripheral = peripheral-vestibular vertigo, somatoform = somatoform vertigo, combination = combination of types.

^3^Self assessment, 5-point-Likert-scale (1 = best health).

^4^Possible range: 0–4 (lower score = less severe and frequent symptoms).

^5^Possible range: 0–100 (lower score = less handicap).

**Table 4:** ICF categories relevant in patients with vertigo (ICF component body functions)

**ICF code**	**ICF label**	**frequency**
***Global mental functions***		
b110	Consciousness functions	4
b114	Orientation functions	1
b126	Temperament and personality functions	3
b1263	Psychic stability	1
b1266	Confidence	2
b1301	Motivation	1
b1302	Appetite	1
b134	Sleep functions	1
b1342	Maintenance of sleep	1
***Specific mental function***s		
b152	Emotional functions	9
b1520	Appropriateness of emotion	2
b156	Perceptual functions	2
b1560	Auditory perception	1
b1561	Visual perception	3
b160	Thought functions	1
***Seeing and related functions***		
b210	Seeing functions	1
***Hearing and vestibular functions***		
b2301	Sound discrimination	1
b2351	Vestibular function of balance	4
b240	Sensations associated with hearing and vestibular function	1
b2400	Ringing in ears or tinnitus	1
b2401	Dizziness	12
b2402	Sensation of falling	3
b2403	Nausea associated with dizziness or vertigo	5
b2405	Aural pressure	1
***Pain***		
b28010	Pain in head and neck	3
***Functions of the cardiovascular system***		
b4200	Increased blood pressure	3
b4201	Decreased blood pressure	1
***Additional functions and sensations of the cardiovascular and respiratory systems***		
b455	Exercise tolerance functions	1
b4550	General physical endurance	1
b4552	Fatiguability	1
b460	Sensations associated with cardiovascular and respiratory functions	2
***Functions related to the digestive system***		
b5106	Regurgitation and vomiting	5
b530	Weight maintenance functions	3
b535	Sensations associated with the digestive system	1
b5350	Sensation of nausea	1
***Movement functions***		
b760	Control of voluntary movement functions	3
b7602	Coordination of voluntary movements	1
b770	Gait pattern functions	8
b780	Sensations related to muscles and movement functions	4
***Functions of the skin***		
b830	Other functions of the skin	2

**Table 5:** ICF categories relevant in patients with vertigo (ICF component activities and participation)

**ICF code**	**ICF label**	**frequency**
***Purposeful sensory experiences***		
d110	Watching	2
d115	Listening	1
***Applying knowledge***		
d166	Reading	2
d170	Writing	1
***General tasks and demands***		
d220	Undertaking multiple tasks	1
d230	Carrying out daily routine	8
d2301	Managing daily routine	1
d2303	Managing one's own activity level	2
d240	Handling stress and other psychological demands	3
***Changing and maintaining body position***		
d4100	Lying down	3
d4102	Kneeling	1
d4103	Sitting	1
d4104	Standing	2
d4105	Bending	3
d415	Maintaining a body position	3
d4154	Maintaining a standing position	4
***Carrying, moving and handling objects***		
d430	Lifting and carrying objects	1
d440	Fine hand use	1
d4401	Grasping	1
d445	Hand and arm use	1
***Walking and moving***		
d450	Walking	8
d4500	Walking short distances	1
d4501	Walking long distances	2
d4502	Walking on different surfaces	3
d4551	Climbing	4
d4552	Running	1
d4554	Swimming	1
d460	Moving around in different locations	5
d4601	Moving around within buildings other than home	1
d4602	Moving around outside the home and other buildings	4
***Moving around using transportation***		
d4702	Using public motorized transportation	3
d4750	Driving human-powered transportation	6
d4751	Driving motorized vehicles	8
d498	Mobility, other specified	1
***Self-care***		
d530	Toileting	3
d5701	Managing diet and fitness	1
d6200	Shopping	5
***Household tasks***		
d630	Preparing meals	2
d640	Doing housework	4
d6400	Washing and drying clothes and garments	1
d6402	Cleaning living area	2
***Caring for household objects and assisting others***		
d6505	Taking care of plants, indoors and outdoors	1
d6506	Taking care of animals	1
d660	Assisting others	1
***Particular interpersonal relationships***		
d730	Relating with strangers	1
d7401	Relating with subordinates	1
d750	Informal social relationships	1
d7600	Parent–child relationships	1
d7602	Sibling relationships	1
d770	Intimate relationships	2
***Work and employment***		
d8451	Maintaining a job	2
d850	Remunerative employment	6
d8502	Full-time employment	1
d855	Non-remunerative employment	1
***Economic life***		
d870	Economic self-sufficiency	1
***Community, social and civic life***		
d910	Community life	2
d9102	Ceremonies	1
d920	Recreation and leisure	2
d9201	Sports	4
d9202	Arts and culture	2
d9205	Socializing	4
d9208	Recreation and leisure, other specified	1

**Table 6:** ICF categories relevant in patients with vertigo (ICF component environmental factors)

**ICF code**	**ICF label**	**frequency**
***Products and technology***		
e110	Products or substances for personal consumption	1
e1100	Food	3
e1101	Drugs	7
e115	Products and technology for personal use in daily living	2
e120	Products and technology for personal indoor and outdoor mobility and transportation	1
e1200	General products and technology for personal indoor and outdoor mobility and transportation	1
e1201	Assistive products and technology for personal indoor and outdoor mobility and transportation	2
e1250	General products and technology for communication	1
e155	Design, construction and building products and technology of buildings for private use	1
***Natural environment and human-made changes to environment***		
e215	Population	1
e2151	Population density	2
e225	Climate	3
e2250	Temperature	1
e245	Time-related changes	1
e2450	Day/night cycles	3
e250	Sound	2
e2500	Sound intensity	3
e255	Vibration	1
***Support and relationships***		
e310	Immediate family	11
e315	Extended family	1
e320	Friends	4
e325	Acquaintances, peers, colleagues, neighbours and community members	1
e355	Health professionals	1
***Attitudes***		
e410	Individual attitudes of immediate family members	2
e420	Individual attitudes of friends	2
e430	Individual attitudes of people in positions of authority	2
e445	Individual attitudes of strangers	5
e450	Individual attitudes of health professionals	4
***Services, systems and policies***		
e515	Architecture and construction services, systems and policies	1
e5200	Open space planning services	1
e5351	Communication systems	1
e540	Transportation services, systems and policies	1
e5402	Transportation policies	1
e5600	Media services	2
e580	Health services, systems and policies	2
e5800	Health services	5
e5801	Health systems	2
e5802	Health policies	4
e590	Labour and employment services, systems and policies	1
e5902	Labour and employment policies	1

Besides the most prominent aspect “dizziness” which was reported by all participants, most participants (9) reported problems within “Emotional functions (b152)” including appropriateness, regulation and range of emotions. Eight participants reported problems related to mobility (including walking and related functions and driving motorized vehicles) and carrying out the daily routine. In addition, almost all participants (11) reported “Immediate family (e310)” as a relevant modifying environmental factor. The next most frequent environmental factor was “Drugs (e1101)”, which was mentioned by seven participants. The results of the analysis of all interviews are shown in Tables 4, 5 and 6.

## Discussion

Our study indicates that patients with vertigo or dizziness consider many different aspects of functioning and health as relevant. Aspects from the component Activities and Participation were most prominently captured, followed by aspects covering Body Functions. Various environmental and personal factors contributed to the multifaceted picture of functioning in vertigo. In our study the ICF turned out to be a useful tool to code various impairments and restrictions caused by vertigo and dizziness and to describe disease specific functioning and quality of life since the majority of concepts in patients’ statements could be linked to the ICF.

Regarding the ICF component Body Functions participants reported, apart from obvious aspects (e.g. sensations associated with hearing and vestibular functions), orientation, temperament and personality functions, confidence, motivation, emotion, sleep, appetite and exercise tolerance as relevant. It is commonly recognized that vertigo has considerable impact on quality of life, but the distinct components are often unclear. Generic instruments such as the SF-36 [26] give but a very general picture of impairment of mental functions. Anxiety and avoidance have been reported as psychological consequences of vertigo [1,27]. Also, emotional problems seem to be associated with vertigo [28]. Depression and somatization as well as increased obsessive-compulsive attitude have been observed [29].

"“(…) It really gets me down.” (b152 Emotional functions, Pt. 6)"

"„Okay, just from the beginning, I wanted to be left alone (…) I didn’t want to go anywhere (…) And when I got such panic attacks … nervous break down… because I don’t want to go outside (…). (b152 Emotional functions Pt. 009)"

"“And at night it is like…sleeping properly… does not work any more.” (b134 Sleeping functions, Pt. 001)"

Of the ICF component Activities and Participation, 62 categories were mentioned as relevant in our study. This underlines how much the pathology interferes with daily life. The reported categories ranged from very basic activities such as watching, listening and reading to more complex items such as managing daily routine, using transportation, self-care and housework:

"“When things pass by quickly… This is what you have to avoid and you actually do avoid… Just like watching TV. I haven’t been watching TV since December.” (d110 Watching, Pt. 001)"

"“I really loved reading. At the moment I can’t really read, because I get dizzy after a little while.” (d166 Reading, Pt. 009)"

"“When you want to go by bus, you have to take a rest at the bus stop…and then you have to get in the bus fast. And you have to get out fast and again… have a rest at the bus stop. Everything gets more complicated.” (d4702 Using public motorized transportation, Pt. 011)"

"“And I am happy to reach the toilet [without a vertigo attack]” (d530 Toiletting, Pt. 012)"

"“It’s worst when I have to do homework, for example when I have to hang curtains…” (d6402 Cleaning living areas, Pt. 008)"

It is obvious that all movements and body positions that are likely to provoke attacks were reported as restricted.

"„As soon as I was standing up… upright… [vertigo attacks occurred]… then walking was really hard (…).“(d4104 Standing, Pt. 006)"

Almost all aspects of social participation were perceived as restricted, namely personal and formal relationships, employment, leisure, sports and community life. This is in line with the work of Neuhauser and colleges who showed in a large, community based study that up to 20% of individuals with vertigo avoid leaving the house [7]. Dizziness is known to impede activities of daily life [30] and to interfere with workplace activities [1]. Because attacks of vertigo are mostly uncontrollable and unpredictable, many patients suffer from the mere anticipation of the attack [31] which may be as restricting as the pathology itself [10].

"„Okay…but… now there is the vertigo (…) when I am standing in front of my customers, or employees… swaying… That doesn’t work!” (d730 Relating with strangers, d7401 Relating with subordinates, Pt. 009)"

"„[At work] I have to prepare these trolley tables all the time. Ist a permanent.. standing, bending, preparing… arround these trolleys… Then bringing them allover the building… four stories… I can’t imagine that I could handle this at the moment.” (d850 remunerative employment, Pt. 001)"

"“I would prefer going to work. But my ENT-doctor says that it’s too risky because of the spells… vomiting, high blood pressure, panic attacks,…” (d850 Remunerative employment, Pt. 001)"

Environmental Factors were reported as interfering and interacting with functioning. Among those were factors of the natural and built environment such as sounds, vibrations, climate, temperature, population density and architectural design. To give an example, paroxysmal positioning vertigo is suspected to be associated with temperature variation and air pollution [32].

"„Yes…, in daily life… crowdy places… where a lot of people… and noisy…This is what I cannot tolerate at the moment.” (e2500 Sound intensity, e2151 Population density, Pt. 001)"

"„And as I’ve said before, when I’m walking, strolling in the sun, I feel that the vertigo is even more extreme.” (e225 Climate, Pt. 008)"

Likewise, family, friends, neighbors, colleagues and their attitudes were reported as relevant environmental factors. They can act as facilitators or barriers for the individual affected by vertigo, since vertigo may change social roles and behaviors, also towards others [33].

"“I have two daughters. They give me a lot of support. And my husband.” (e310 Immediate family, Pt. 001)"

"„They, the people next to me, or acquaintances, too… When we went out for diner or to cinema… They knew that I suffer from vertigo attacks and that gave me the freedom… when I recognized: Oh! There is.... Then I could call them to help me: „Bring me to my car“(…).” (e420 Individual attitudes of friends, Pt. 012)"

In general, the ICF turned out to be a useful tool to describe the situation, determinants and consequences of vertigo. Only few concepts reported by the patients could not be coded with ICF categories. Most of them were related to personal factors, mostly coping strategies. As an example, some patients reported to visit only places which are save if a vertigo attack occurs, such as buildings that have staircases with handlebars, or places where they can lay down during a vertigo attack. Other patients reported to schedule trips or shopping more carefully. Coping strategies and resilience are reported in the literature as major determinants of symptom severity [34].

### Methodological considerations

It was not the intention of our study to draw generalizing conclusions on experiences towards functioning and health of vertigo patients, or to report impact in various subgroups, or to compare them amongst others. We did include patients with different types of diagnoses in our study to ensure covering the widest range of experiences. The qualitative approach should always be the primary method to get a first idea about relevant aspects of functioning. As in quantitative studies, validity of this largely depends on the choice of participants. Participants should represent the typical range for which subsequent studies should be representative. The results of our study should provide a first selection of patient-relevant items which have to prove its validity in terms of frequency and discrimination before being implemented in further applications, such as future studies or clinical issues.

### Limitations

Some limitations have to be mentioned. On the one side, selection of patients for the interviews might be biased towards individuals with milder disease who are capable to undergo an interview procedure. On the other side, patients seen in a specialized tertiary care dizziness clinic may be selected towards those with more severe symptoms or more chronic conditions. However, our findings have high face validity and are in line with the studies conducted in this field.

Altogether, our study gives a first impression from the patients' perspective using the ICF regardless of potential selection bias.

## Conclusions

From the patients’ perspective, vertigo has impact on multifaceted aspects of functioning and disability, mainly body functions and activities and participation. Modifying contextual factors have to be taken into account to cover the complex interaction between the health condition of vertigo on the individuals’ daily life. While there are generic and disease-specific instruments to measure Quality of life which do encompass several of the aspects found in our study, the ICF offers a broader perspective and a full understanding of all the factors involved at individual and health care level. The ICF has, therefore, a great potential to comprehensively describe functioning in vertigo for the assessment of burden of disease and treatment success. The results of this study will contribute to developing standards for the measurement of functioning, disability and health relevant for patients suffering from vertigo.

## Abbreviations

ICF, International classification of functioning disability and health; WHO, World health organization; SF-36, Short form (SF)-36 health survey; IFBLMU, Integrated center for research and treatment of vertigo balance and ocular motor Disorders; DHI, Dizziness handicap inventory; VSS, Vertigo symptoms scale.

## Competing interests

The authors declare that they have no competing interests.

## Authors’ contributions

MM and EG designed the study. ES carried out the interviews. MM supervised data collection. MM, RS and ES analyzed the data. All Authors interpreted the results and contributed in drafting the manuscript. All authors read and approved the final manuscript.
